# Core Circadian Clock Proteins as Biomarkers of Progression in Colorectal Cancer

**DOI:** 10.3390/biomedicines9080967

**Published:** 2021-08-06

**Authors:** María I. Aroca-Siendones, Sara Moreno-SanJuan, Jose D. Puentes-Pardo, Michela Verbeni, Javier Arnedo, Julia Escudero-Feliu, María García-Costela, Adelina García-Robles, Ángel Carazo, Josefa León

**Affiliations:** 1 Biobank of the Public Health System of Andalusia, 18016 Granada, Spain; ines.aroca@juntadeandalucia.es; 2 Biosanitary Research Institute of Granada (ibs.GRANADA), 18012 Granada, Spain; josedavidpupa@correo.ugr.es (J.D.P.-P.); juliaescuderofeliu@gmail.com (J.E.-F.); gmaria92@correo.ugr.es (M.G.-C.); adelingrobles@hotmail.com (A.G.-R.); ext.acarazo@ugr.es (Á.C.); 3 Cytometry and Microscopy Research Service, Biosanitary Research Institute of Granada (ibs.GRANADA), 18012 Granada, Spain; sara.moreno@ibsgranada.es; 4 Department of Pharmacology, Faculty of Pharmacy, University of Granada, 18011 Granada, Spain; 5 Department of Computer Science and Artificial Intelligence, University of Granada, 18071 Granada, Spain; michelav@decsai.ugr.es; 6 Bioinformatics Service, Instituto de Investigación Biosanitaria de Granada, ibs.GRANADA, 18012 Granada, Spain; jarnedo@ibsgranada.es; 7 Clinical Management Unit of Digestive Disease and UNAI, San Cecilio University Hospital, 18016 Granada, Spain

**Keywords:** colorectal cancer, core circadian clock, metachronous metastasis, local recurrence, overall survival, disease free survival

## Abstract

Colorectal cancer (CRC) is one of the most common tumours in developed countries. Although its incidence and mortality rates have decreased, its prognosis has not changed, and a high percentage of patients with CRC develop relapse (metachronous metastasis, MM, or local recurrence, LR) during their disease. The identification of these patients is very important for their correct management, but the lack of prognostic markers makes it difficult. Given the connection between circadian disruption and cancer development and progression, we aimed to analyse the prognostic significance of core circadian proteins in CRC. We measured the expression of PER1-3, CRY1-2, BMAL1 and NR1D2 in a cohort of CRC patients by immunohistochemistry (IHC) and analysed their prognostic potential in this disease. A low expression of PER2 and BMAL1 was significantly associated with metastasis at the moment of disease diagnosis, whereas a high expression of CRY1 appeared as an independent prognostic factor of MM development. A high expression of NR1D2 appeared as an independent prognostic factor of LR development after disease diagnosis. Moreover, patients with a low expression of BMAL1 and a high expression of CRY1 showed lower OS and DFS at five years. Although these markers need to be validated in larger and different ethnic cohorts, the simplicity of IHC makes these proteins candidates for personalizing CRC treatment.

## 1. Introduction

Colorectal cancer (CRC) is one of the most common tumours in developed countries and one of the leading causes of death in the world. There are gender differences in the incidence rates, with CRC being the third most common cancer in men, after lung and prostate cancer and the second most common in women, after breast cancer [1]. CRC incidence and mortality rates have decreased over the last two decades due to recent advances in prevention and detection [2]. However, the prognosis of CRC has currently not changed, and a high percentage of patients with CRC will have a relapse after treatment [3]. In fact, more than 50% of patients will develop liver metastases during the course of their disease (metachronous metastasis, MM), which represent the main cause of morbidity and mortality [4]. Local recurrence (LR) at the first site of disease, is much less common, constituting 10% to 20% of all recurrences [5].

A great effort has been done in order to identify patients with unfavourable prognosis that may benefit from adjuvant therapy. From the classical TNM staging system [6], several attempts to improve cancer classification have been proposed [7,8,9,10]. However, these methods have mainly focused on ‘omics’ and massive approaches that mask intra-tumour and inter-patient heterogeneity and involve high costs, avoiding large-scale clinical application [11]. Promising strategies centred their attention on the tumour microenvironment [12] or the central hallmarks of cancer [13], which are present in almost all tumours regardless of the underlying molecular changes. Cancer cells possess uncontrolled proliferation resulting from the aberrant activity of various cell cycle proteins [14]. Since the circadian clock and the cell cycle systems are robustly phase-coupled in a bidirectional manner [15], the molecular components of the circadian clock could be considered as prognostic markers.

Circadian rhythms are a class of endogenous biological rhythms with a period of about 24 h [16], synchronized by the suprachiasmatic nucleus (SCN) with the light/dark cycle of the environment [17]. Other existing peripheral clocks (heart, skin or colon) generate fluctuations regardless of the SCN, although all are coordinated by it [18,19]. In molecular terms, circadian rhythms are generated by transcription–translation feedback (TTFL) loops in which CLOCK and BMAL1 dimers act as transcription factors that modulate the expression of PER1/2/3, CRY1/2 and other genes. An additional loop formed by ROR and NR1D1/2 regulates the main cycle [18,20]. In addition, a machinery of post-translational modifications is involved in the regulation of the correct ticking of the clock, including phosphorylation, acetylation/deacetylation, SUMOylation or methylation [21].

Many studies have demonstrated the interplay between circadian rhythms dysregulation and the initiation and progression of cancer [22]. In addition, the effectiveness of treatments in various types of cancer depends on the circadian clock [23]. Therefore, the integration of circadian biology into cancer research offers new options for the prevention, diagnosis and treatment of this disease [21,24,25].

Specifically, the connection between circadian disruption and CRC development, progression, incidence and resistance to treatments has been extensively studied [26,27,28]. However, these results have not yet been transferred to the clinic, since this relationship is in some cases controversial [22]. In this context, we aimed to identify the expression patterns of circadian clock proteins by immunohistochemistry, particularly PER1/2/3, CRY1/2, BMAL1 and NR1D2 in a cohort of patients with CRC and examine their role in the progression of the disease and outcome of patients. We found that CRY1 could be a potential marker of MM, while NR1D2 was associated with LR, which could be translated to the clinic to improve the management of these patients.

## 2. Materials and Methods

### 2.1. Patients and Samples

The study has been approved by the Research Ethic Committee of Granada (Andalusia, Spain) (PI-0677-2013) and has been carried out in compliance with the guidelines of the Declaration of Helsinki. All patients gave informed consent to participate in the study. The tumour tissues were obtained in the surgical intervention of the primary tumour between 9.00 a.m. and 1.00 p.m. and provided by the Andalusian Public Health System Biobank.

The following clinic-pathologic data were collected for each patient: age, gender, general location (rectal or colonic), specific location (ascending-colon, hepatic flexure, transverse colon, splenic flexure, descending colon, sigmoid colon and rectum), number of nodes removed/number of nodes with metastasis, date and type of treatment (surgery, chemotherapy and/or radiotherapy), evaluation of response to treatment (relapse or metastasis) and, finally, data relative to overall survival (OS) and disease free survival (DFS) were recorded at the end of the study. Tumour samples were classified according to the degree of differentiation based on the criteria of the World Health Organization [29]. The stage was determined according to the American Joint Committee on Cancer Staging System [30].

Patients were recruited between 2004 and 2014 and followed until November 2018 (maximum 14.2 years). Patients treated with neoadjuvant therapies, diagnosed with hereditary cancer or a previous cancer, treated or not, were discarded. Under these excluding criteria, 258 cases of embedded paraffin tissue samples of colorectal cancer (carcinoma and adenocarcinoma) and 66 cases of normal colon tissue used as control samples were considered for the study. Tumour recurrence at nonregional sites, such as liver or lung, was recorded as metachronous metastasis (MM) and only in stage II and III patients. Local recurrence (LR) was recorded regardless of the presence of metastatic disease [31]. The clinicopathological characteristics of tumour samples are described in Table 1.

### 2.2. IHC Analysis

Paraffin-embedded tissues were sectioned continuously at a thickness of 3 μm and heated for 1 h at 60 °C. The sections were then deparaffinized using xylene at 37 °C for 20 min and rehydrated with a series of graded alcohol and distilled water. The tissue slides were then treated with 3% hydrogen peroxide in methanol for 20 min at 37 °C to block endogenous peroxidase activity. The sections were subsequently immersed in 10 mM citrate buffer (pH 6.0), microwaved for antigenic retrieval and allowed to cool to room temperature. This treatment was followed by incubation with a primary antibody, Anti-PER3 (Q-16; 1:50 dilution) and Anti-PER2 (19-J6; 1:50 dilution) by Santa Cruz Biotechnology (Dallas, TX, USA), Anti-PER1 (ab3443; 1:100 dilution), Anti-CRY1 (ab54649; 1:100 dilution), Anti-CRY2 (ab38872; 1:50 dilution) and Anti- NR1D2 (NR1D2) (ab41940; 1:25 dilution) by Abcam (Cambridge, UK), and Anti-BMAL1 (1C11; 1:50 dilution) by Novus Biologicals (Centennial, CO, USA) in a humidified container at 4 °C. The specific conditions for each antibody are included in Table S1. The tissue slides were washed three times with PBS, incubated with the corresponding secondary anti-bodies, either an anti-rabbit (1:200 dilution) or anti-mouse (1:200 dilution) by Roche (Basel, Switzerland), at 37 °C for 30 min, and then thoroughly washed three times with PBS. The sections were developed with diaminobenzidine tetrahydrochloride (DAB), counterstained with haematoxylin and mounted with permanent medium (DPX). Negative second-layer controls were included in each assay, omitting the primary antibody, to rule out false positives due to nonspecific reactions of the secondary antibody with the tissue. Once the optimal protocol for each antibody was determined, the immunohistochemical staining was carried out using the ROCHE “Discovery Ultra Benchmark” automatic immunotec (Basel, Switzerland).

### 2.3. Evaluation of Staining

The intensity and the percentage of expression for each marker were semi-quantitatively and independently evaluated by two independent researchers who were blinded to the patient data. All cases where inter-observer disagreement occurred were discussed together with a third observer until agreement was reached on the final expression score. The results were informed according to the percentage of cells stained as: 0 < 5%, 1 = 6–25%, 2 = 26–50%, 3 = 51–75%, 4 > 75% of cells stained. Similarly, the intensity of staining was scored as: 0 = no staining, 1 = weak, 2: moderate and 3 = strong staining. The two scales were multiplied to obtain the final immunoreactive (IRS) score scale from 0 to 12, as described previously [32,33]. The staining scores of the tissue controls in each microarray slide were pre-evaluated as a quality control.

### 2.4. Statistical Analysis

Low and high IRS values for each protein were stablished by receiver operator characteristic (ROC) analysis [34] for DFS at 3 and 5 years after disease diagnosis (Table 2). All proteins studied showed the same optimal cut point (OCP) value at those times. In some samples, it was not possible to evaluate all proteins.

To establish the relationship between the proteins studied and the clinicopathological features of patients, they were dichotomized as follows: T stage (early (T1 + T2) or late (T3 + T4)), N stage (N0 (no lymph node involvement) or >N0 (any lymph node involvement)), M stage (M0 (no metastasis presence) or M1 (any presence of metastasis)), TNM stage (early (I + II) or advanced (III + IV)) and survival (death due to CRC or censored (lost to follow-up, alive or death from other causes)).

A bivariate analysis was performed using the χ^2^ and the Fisher’s exact tests. The Kaplan–Meier method was used to determine the cumulative probability of OS and DFS, and the differences were evaluated using Log-rank tests. Prognostic factors were evaluated using multivariate analysis (Logistic regression or Cox proportional hazards regression model). The tests were carried out with 95% confidence, considering significant those with a *p* value below 0.05. All statistical analyses were performed using SPSS software version 18.0 (SPSS Inc., Chicago, IL, USA) and according to REMARK criteria [35]. The mosaic plots were done using Orange software version 3.29.3 [36]. The Mosaic plot is used for visualizing data from two or more qualitative variables. It provides the user with the means to recognize relationships between different variables more efficiently [37,38].

## 3. Results

### 3.1. Expression of Core Circadian Clock Proteins in Tissues from Healty Subjects and CRC Patients

**Figure 1:**
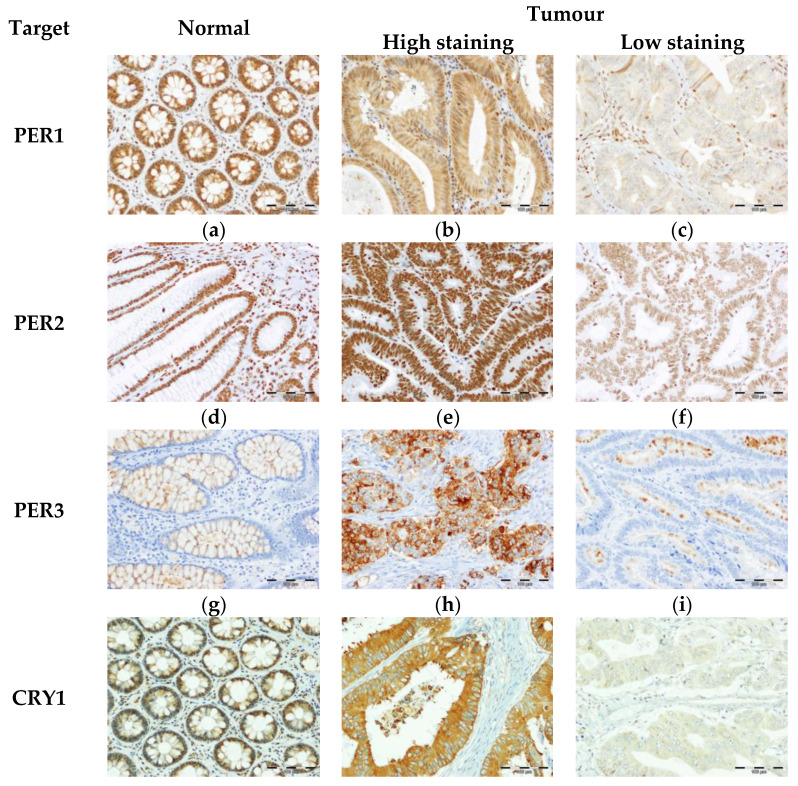

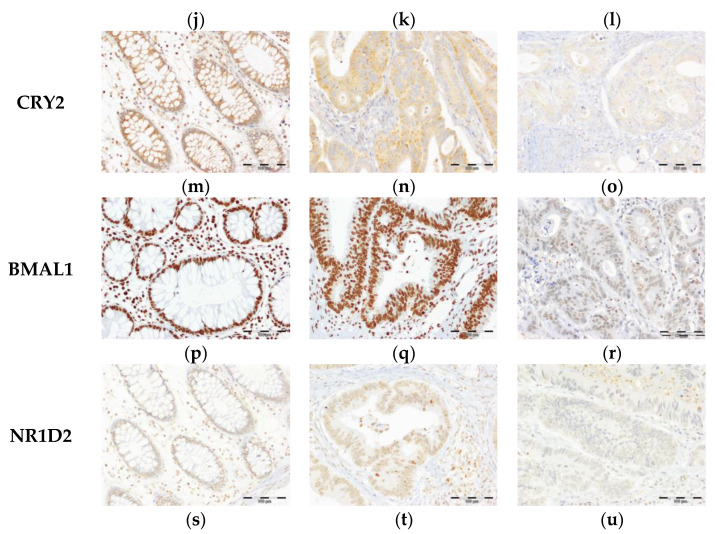
IHC staining of PER1 (**a**–**c**), PER2 (**d**–**f**), PER3 (**g**–**i**), CRY1 (**j**–**l**) CRY2 (**m**–**o**), BMAL1 (**p**–**r**) and NR1D2 (**s**–**u**) in normal colonic mucosa (**a**,**d**,**g**,**j**,**m**,**p**,**s**) and primary tumours of CRC with high (**b**,**e**,**h**,**k**,**n**,**q**,**t**) and low (**c**,**f**,**i**,**l**,**o**,**r**,**u**) staining. Magnification: 200×.

We analysed the expression of PER1/2/3, CRY1/2, BMAL1 and NR1D2 in normal colonic tissues from control donors and in CRC samples obtained from patients by IHC (Figure 1).

As shown in Table 3, all clock-related proteins were significantly more expressed in normal mucosa than in tumour tissues, except for BMAL1.

### 3.2. Association of the Core Circadian Clock Proteins with Clinico-Pathological Characteristics of CRC Patients

Low and high IRS values for each protein were stablished trough OCP data obtained as described in the Materials and Methods section, and were used to analyse the relationship between their expression and the clinicopathological characteristics of the patients with CRC included in the study. As shown in Table 4 and Table 5, men presented a higher expression of PER2 (*p* = 0.016) than women. Interestingly, well differentiated tumours correlated significantly with a high expression of PER2 (*p* = 0.009), which gradually decreased with the differentiated state of tumours. The propagation (metastasis) to distant sites (M) at the moment of the disease diagnosis appeared in tumours with low levels of BMAL1 (*p* = 0.004) and PER2 (*p* = 0.037). Tumour progression (TNM stage) correlated with PER1 (*p* = 0.020), and early-stage tumours (stage I + stage II) showed a higher expression of such proteins than those of advanced stage (stage III + stage IV).

### 3.3. CRY1 as a Prognostic Factor of MM in CRC

A very important issue in the management of patients with CRC is the possibility of the development of MM after disease diagnosis. Therefore, we have analysed the relationship of the core circadian clock proteins expression with this variable. The appearance of MM correlated significantly with a high expression of CRY1 in all cases (*p* = 0.003), when it appears at 3 years after disease diagnosis (*p* = 0.017) and also if it appears at 5 years after disease diagnosis (*p* = 0.008) (Table 6). None of the other proteins analysed were related to the appearance of MM (Table S2).

The logistic regression analysis showed CRY1 and adjuvant therapy as independent prognostic factors for MM development after 3 and 5 years of disease diagnosis (Table 7).

**Figure 2:**
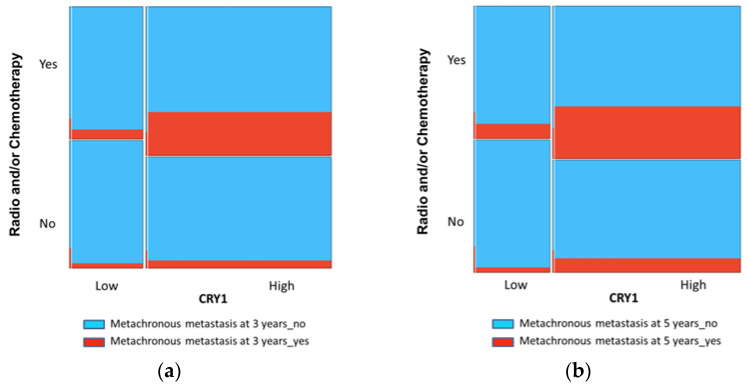
Mosaic plots showing the number of patients who have developed MM at 3 (**a**) and 5 (**b**) years after the diagnosis of CRC.

In addition, mosaic plots confirm an increase in the number of patients developing MM at 3 and 5 years when they received therapy (radiotherapy and/or chemotherapy) and had high CRY1 expression in tumours (Figure 2).

### 3.4. Proteins of the Core Circadian Clock as Prognostic Factors of LR in CRC

Although LR after disease diagnosis is less frequent in CCR than MM, it is also very important to know the possibility of its development to apply personalized medicine in these patients. The appearance of LR correlated significantly with a high expression of NR1D2 in all cases (*p* = 0.031), when it appears at 3 years after disease diagnosis (*p* = 0.015) and also at 5 years after disease diagnosis (*p* = 0.042) (Table 8). None of the other proteins analysed were related to the appearance of LR (Table S3).

Logistic regression analysis showed NR1D2 as an independent prognostic factor for LR after 3 and 5 years of disease diagnosis (Table 9).

### 3.5. Proteins of the Core Circadian Clock as Prognostic Factors of Survival in CRC

**Figure 3:**
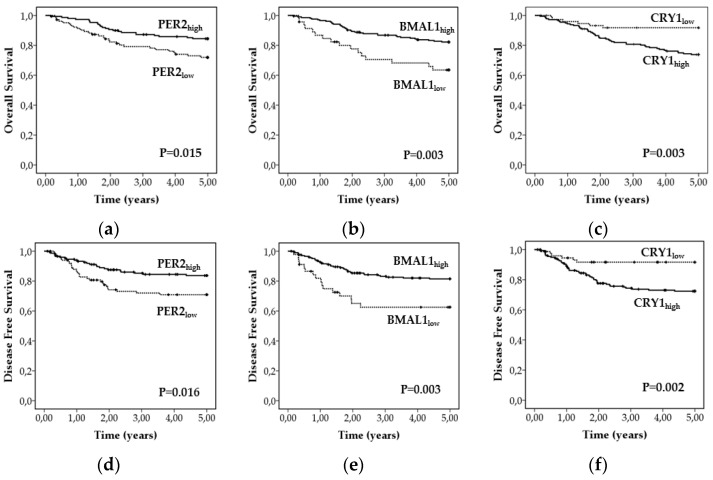
Kaplan–Meier curves depicting OS and DFS according to expression patterns of PER2 (**a**,**d**), BMAL1 (**b**,**e**) and CRY1 (**c**,**f**). *p* values were calculated with the log-rank test.

We compared the effect of circadian core proteins’ expression on 5-year overall survival (OS) and disease-free survival (DFS) in our cohort of CRC patients. As shown in Figure 3, high PER2 and BMAL1 expression was associated with a significantly better OS (χ2 = 5.888, *p* = 0.015 and χ2 = 8.875, *p* = 0.003, respectively) and DFS (χ2 = 9.051, *p* = 0.016 and χ2 = 9.051, *p* = 0.003, respectively) (Figure 3a,b,d,e). On the contrary, high CRY1 expression was associated with a significantly worse OS and DFS in our cohort of patients (χ2 = 8.820, *p* = 0.003 and χ2 = 9.551, *p* = 0.002, respectively) (Figure 3c,f).

Further, the multivariate Cox regression for survival analysis showed CRY1 and BMAL1 expression as independent prognostic factors for OS and DFS in patients with CRC (Table 10).

## 4. Discussion

One of the most challenging problems in oncology is the decision-making process in relation to the treatment of the patients, as the survival outcomes vary even in patients with similar clinical or pathologic features. A very important factor determining survival in CRC is the development of MM during the 3–5 years after disease diagnosis, that can affect as much as one half of patients. In this sense, the discovery of new prognostic biomarkers may enable personalized cancer therapies. In this study, we found that low expressions of PER2 and BMAL1 were significantly associated with the presence of metastasis at the moment of disease diagnosis, whereas a high expression of CRY1 was significantly associated with the development of MM after 3 and 5 years of disease diagnosis. More importantly, CRY1 appeared as an independent prognostic factor of MM development, having received adjuvant therapy (chemo and/or radiotherapy) in CRC patients. Although LR is less important in CRC, we found that a high expression NR1D2 appeared as an independent prognostic factor of LR development after disease diagnosis. Moreover, patients with a low expression of BMAL1 and a high expression of CRY1 showed lower OS and DFS at five years, and these proteins are independent prognostic factors for survival in our cohort of patients.

In our study cohort, we found a significant underexpression of PER1/2/3, CRY1/2 and NR1D1 proteins in CRC versus normal tissue, indicating an important role of these genes in colorectal carcinogenesis. This aspect has been extensively reported in a wide range of cancers, including CRC [22,39,40,41]. Polymorphic variants of these circadian genes might contribute to an individual’s risk of developing cancer [42,43,44,45,46]. The interactions between colon cancer cells and tumour-associated fibroblasts can also be responsible for the molecular clockwork disruption, which enhances malignant phenotypes on cancer cells [47]. Other findings showed a feed-back loop between the circadian clock and epigenetic machinery in cancer [25,48,49].

Like other core circadian clock proteins, there are discrepancies in the literature regarding the expression of CRY1 in tumour tissue versus normal mucosa. Previous studies have shown a decrease in CRY1 in tumours compared to normal mucosa [41], which would agree with our results. However, other authors have found the opposite [50]. It is very important to note that the expression of CRY1 changes throughout the colonic tract [41,48] and is also related to gender [49]. Therefore, the expression of CRY1 is conditioned by these two aspects and would explain the differences found between reports.

Paradoxically, we found that patients with a higher expression of CRY1 in their tumours showed lower OS and DFS, probably because these patients also showed an increased risk of developing MM at 3 and 5 years after diagnosis of the disease, which could be used as an advantage for the management of these patients. Cry1 mRNA overexpression has been previously associated with poor OS in CRC [50,51], mainly in elderly subjects, female patients and cancers located at the transverse colon [51]. At the molecular level, mutations of the Cry1 gene in mice cause a low expression of CRY1 protein and the down-regulation of c-MYC [52], which is essential for colorectal tumourigenesis [53]. Other studies evidenced that CRY1 modulates the ATR-mediated DNA damage repair, increasing the survival of cells [54].

LR has been classically associated with viable tumour cells that remain in situ after tumour resection. This may be due to a poor operative technique or to a more aggressive biology of tumours where viable cells have escaped the limits of resectability [31,55]. More recently, tumour factors such as locally aggressive disease, obstruction or multiple positive lymph nodes have been related with high rates of LR, rather than the adherence to oncologic surgical principles in colon cancer resection [31]. We identified a high expression of NR1D2 in the tumour as an independent prognostic factor for LR development. Similarly to CRY1, although the expression of NR1D2 was reduced in tumour tissue compared to normal mucosa, patients with a higher expression of this protein also had an increased risk of developing LR. NR1D2 is a variant of NR1D1 and both proteins have described redundant functions in regulating circadian rhythm, metabolism and inflammatory response [25]. However, whereas NR1D2 is the major variant in various human cancer cells, NR1D1 is more abundant in normal tissues [56]. NR1D2 regulates glioblastoma cell proliferation and motility [57] and accelerates hepatocellular carcinoma progression by driving the epithelial-to-mesenchymal transition [58]. Recently, it has also been implicated in the mechanisms of treatment resistance in prostate cancer [59].

It is interesting that in our study patients who have received treatment (chemo and/or radiotherapy) have a higher risk of developing MM than those patients who have not received it. Resistance to anticancer drugs may occur prior to treatment, involving pre-existing resistance factors in tumour cells, or it may be acquired during the treatment of tumours due to the induction of adaptive responses. In addition, due to the high degree of tumour heterogeneity, drug resistance may also result from the therapy-induced selection of a drug-resistant tumour subpopulation, such as cancer stem cells (CSCs) [60]. Indeed, this tumour subpopulation is responsible for tumour initiation and development, metastasis, and the mentioned resistance to antitumour treatment [61,62]. Cellular plasticity and the microenvironment, among other factors, seem to protect CSCs, thus compromising therapeutic efficacy [61].

Taken together, we found that a low expression of BMAL1 and a high expression of CRY1 are markers of survival in CRC. Furthermore, CRY1 and NR1D2 overexpression can be used as biomarkers for MM and LR, respectively, in this disease. Although these markers need to be validated in larger and different ethnic cohorts and prospective studies are warranted before using them in the clinic, the simplicity of immunostaining and assessment by IRS makes these proteins interesting candidates for personalizing CRC treatment.

## Figures and Tables

**Table 1:** Characteristics of patients included in the study.

		Frequency (N)	Percentage (%)
**Gender**	Man	154	59.7
	Woman	114	40.3
**Age ^a^**	≤71	144	55.8
	>71	114	44.2
**Organ**	Colon	215	83.3
	Recto	43	16.7
**Grade of differentiation**	Well differentiated	88	34.0
	Moderately differentiated	140	54.3
	Poorly differentiated	29	11.7
**T stage**	T1	4	1.6
	T2	29	11.2
	T3	183	70.9
	T4	42	16.3
**N (TNM classification)**	N0	135	52.3
	N1	77	29.8
	N2	44	17.9
**M (TNM classification)**	M0	209	81.0
	M1	45	17.4
	Mx	4	1.6
**Stage**	Stage I	26	11.7
	Stage II (IIA-IIB)	97	36.0
	Stage III (IIIA-IIIB-IIIC)	87	33.7
	Stage IV	45	18.6
**Metachronous metastasis**	No	170	75.5
	Yes	56	25.5
**Local recurrence**	No	190	75.2
	Yes	27	24.8
**Radiotherapy**	No	221	88.4
	Yes	29	11.6
**Chemotherapy**	No	101	41.9
	Yes	140	58.1

^a^ Age was dichotomized by the median.

**Table 2:** OCP obtained for the core circadian clock proteins analysed.

	OCP	Low	High
**PER1**	3	0–3	4–12
**PER2**	5	0–5	6–12
**PER3**	1	0–1	2–12
**CRY1**	3	0–3	4–12
**CRY2**	2	0–2	3–12
**BMAL1**	6	0–6	7–12
**NR1D2**	1	0–1	2–12

**Table 3:** Expression of circadian clock-related proteins in normal and tumour colorectal samples.

	Normal ^a^ (N = 66)	Tumour ^a^ (N = 258)	*p*
**PER1**	8.0 (7.0–10.0)	3.0 (2.0–4.0)	<0.001
**PER2**	12.0 (12.0–12.0)	5.3 (4.0–7.9)	<0.001
**PER3**	4.5 (3.4–7.0)	0.0 (0.0–0.0)	<0.001
**CRY1**	8.0 (6.0–8.0)	4.0 (2.0–5.0)	<0.001
**CRY2**	9.0 (8.0–11.0)	5.3 (4.0–7.5)	<0.001
**BMAL1**	8.0 (5.0–12.0)	8.0 (6.7–10.7)	0.663
**NR1D2**	6.0 (4.0–8.0)	0.3 (0.0–2.0)	<0.001

^a^ Data are represented as median  ±  interquartile range.

**Table 4:** Relationship between expression levels of circadian proteins and clinicopathologic features of the individuals included in the study.

		PER1			PER2			PER3		
		Low	High	*p* ^a^	Low	High	*p* ^a^	Low	High	*p* ^a^
**Age**	**≤71**	63 (44.1)	80 (55.8)	ns	59 (41.1)	84 (58.9)	ns	132 (91.8)	12 (8.2)	ns
	**>71**	46 (40.0)	69 (60.0)		43 (38.7)	72 (61.3)		108 (93.3)	7 (6.7)	
**Gender**	**Men**	61 (39.2)	95 (60.8)	ns	52 (33.1)	103 (66.9)	0.016	142 (91.3)	14 (8.7)	ns
	**Women**	48 (46.7)	54 (53.3)		50 (48.1)	53 (50.9)		98 (94.4)	6 (5.6)	
**GD ^b^**	**WD**	41 (36.4)	47 (63.6)	ns	23 (27.3)	64 (62.7)	0.005	80 (90.9)	8 (9.1)	ns
	**MD**	56 (34.3)	84 (65.7)		62 (45.1)	78 (54.9)		131 (91.5)	10 (8.5)	
	**PD**	11 (42.9)	17 (57.1)		16 (53.3)	13 (47.7)		27 (86.2)	2 (13.8)	
**T Stage**	**T1-T2**	11 (45.5)	22 (54.5)	ns	11 (36.4)	22 (63.6)	ns	32 (97.0)	1 (3.0)	ns
	**T3-T4**	97 (63.4)	126 (36.6)		90 (54.9)	134 (45.1)		207 (88.5)	19 (11.5)	
**N Stage**	**N0**	50 (31.1)	85 (68.9)	ns	48 (34.4)	88 (65.6)	ns	125 (91.9)	11 (8.1)	ns
	**N1-N2**	58 (43.4)	63 (56.6)		53 (44.9)	68 (55.1)		110 (89.4)	13 (10.6)	
**M Stage**	**M0**	81 (39.3)	125 (60.7)	ns	77 (35.3)	132 (64.7)	0.040	193 (91.5)	17 (9.5)	ns
	**M1**	24 (57.4)	22 (42.6)		24 (52.2)	21 (47.8)		42 (93.5)	3 (6.5)	
**TNM**	**I-II**	42 (34.4)	81 (65.6)	0.015	43 (34.7)	81 (85.3)	ns	115 (92.7)	10 (7.3)	ns
	**III-IV**	65 (48.9)	67 (51.1)		57 (43.8)	73 (56.2)		121 (96.3)	10 (3.7)	

^a^ χ^2^ or Fisher’s exact tests; ^b^ Grade of differentiation. WD: well differentiated, MD: moderately differentiated, PD: poorly differentiated. ns: non significant.

**Table 5:** Relationship between expression levels of circadian proteins CRY1-2, BMAL1 and NR1D2 and clinicopathologic features of the individuals included in the study.

		CRY1			CRY2			BMAL1			NR1D2		
		Low	High	*p* ^a^	Low	High	*p* ^a^	Low	High	*p* ^a^	Low	High	*p* ^a^
**Age**	**≤71**	44 (30.8)	100 (69.2)	ns	11 (7.5)	133 (92.5)	ns	30 (21.4)	113 (78.6)	ns	86 (60.3)	57 (39.7)	ns
	**>71**	28 (26.1)	87 (73.9)		5 (5.0)	111 (95.0)		17 (12.4)	97 (88.6)		63 (53.3)	53 (47.7)	
**Gender**	**Men**	40 (32.2)	116 (76.8)	ns	6 (3.8)	150 (96.3)	ns	23 (18.3)	130 (87.1)	ns	88 (84.5)	67 (15.5)	ns
	**Women**	32 (30.1)	71 (69.9)		10 (10.3)	94 (89.7)		24 (29.1)	80 (70.9)		61 (81.6)	43 (18.4)	
**GD ^b^**	**WD**	25 (37.5)	63 (62.5)	ns	3 (12.5)	85 (87.5)	ns	15 (46.5)	71 (53.5)	ns	44 (52.3)	44 (47.7)	ns
	**MD**	37 (40.0)	103 (60.0)		11 (16.3)	130 (83.7)		23 (51.1)	118 (48.9)		87 (63.8)	54 (36.2)	
	**PD**	11 (48.3)	18 (51.7)		2 (6.9)	27 (93.1)		8 (82.1)	20 (17.9)		17 (67.9)	11 (32.1)	
**T Stage**	**T1-T2**	11 (54.5)	22 (45.5)	ns	1 (9.1)	32 (90.9)	ns	3 (12.1)	30 (87.9)	ns	17 (42.4)	18 (57.6)	ns
	**T3-T4**	61 (75.1)	164 (24.9)		15 (14.6)	211 (85.4)		43 (25.6)	180 (74.4)		131 (50.7)	94 (49.3)	
**N Stage**	**N0**	127 (36.3)	10 (63.7)	ns	5 (3.3)	131 (96.7)	ns	20 (13.3)	115 (86.7)	ns	80 (61.0)	57 (39.0)	ns
	**N1-N2**	112 (45.5)	10 (54.5)		11 (6.9)	112 (93.1)		26 (16.9)	95 (83.1)		68 (57.4)	53 (42.6)	
**M Stage**	**M0**	60(35.6)	149 (64.4)	ns	14 (6.2)	196 (93.8)	ns	31 (15.1)	176 (85.9)	0.005	121 (58.2)	87 (41.8)	ns
	**M1**	11 (47.8)	34 (52.2)		2 (4.1)	43 (95.9)		15 (30.4)	30 (69.6)		26 (56.5)	20 (43.5)	
**TNM**	**I-II**	33 (26.6)	91 (63.4)	ns	4 (3.2)	120 (96.8)	ns	17 (13.8)	106 (86.2)	ns	74 (59.7)	50 (40.3)	ns
	**III-IV**	38 (29.0)	93 (71.0)		12 (9.1)	121 (90.9)		29 (22.3)	101 (77.7)		73 (55.7)	58 (44.3)	

^a^ χ^2^ or Fisher’s exact tests; ^b^ Grade of differentiation. WD: well differentiated, MD: moderately differentiated, PD: poorly differentiated. ns: non significant.

**Table 6:** Relationship between expression levels of circadian proteins and development of MM after disease diagnosis of individuals included in the study.

		CRY1		
		Low	High	*p* ^a^
**All patients**	**No**	52 (33.3)	104 (66.7)	0.003
	**Yes**	4 (9.8)	37 (90.2)	
**3 years after disease diagnosis**	**No**	51 (31.3)	112 (68.7)	0.017
	**Yes**	3 (10.0)	27 (90.0)	
**5 years after disease diagnosis**	**No**	49 (32.9)	100 (67.1)	0.008
	**Yes**	4 (10.8)	33 (89.2)	

^a^ χ^2^ or Fisher’s exact tests.

**Table 7:** Results of logistic regression for metastasis development within 3 and 5 years after disease diagnosis.

3 Years after Disease Diagnosis			
Independent Variables		OR ^a^ [95% CI ^b^]	*p* Value
**Intercept**			<0.0001
**Age**	(>71 vs ≤71)	1.12 [0.44, 2.90]	0.811
**Gender**	(man vs. woman)	2.013 [0.78, 5.19]	0.148
**T stage**	(T3 + T4 vs. T1 + T2)	1.69 [0.34, 8.34]	0.520
**N stage**	(N1 + N2 N0 vs)	1.14 [0.49, 2.80]	0.780
**Adjuvant Therapy**	(Yes vs. No)	4.30 [1.29, 14.29]	0.017
**CRY1**	(High vs. Low)	3.81 [1.07, 13.57]	0.039
**5 years after disease diagnosis**			
**Independent variables**		**OR [95% CI]**	***p*** **value**
**Intercept**			<0.0001
**Age**	(>71 vs ≤71)	1.37 [0.57, 3.27]	0.479
**Gender**	(man vs. woman)	1.43 [0.63, 3.26]	0.395
**T stage**	(T3 + T4 vs. T1 + T2)	0.97 [0.28, 3.39]	0.965
**N stage**	(N1 + N2 N0 vs)	1.19 [0.51, 2.80]	0.683
**Adjuvant Therapy**	(Yes vs. No)	3.72 [1.31, 10.60]	0.014
**CRY1**	(High vs. Low)	3.87 [1.26, 11.88]	0.018

^a^ Odd Ratio; ^b^ Confidence Intervals.

**Table 8:** Relationship between expression levels of circadian proteins and development of LR after disease diagnosis of patients included in the study.

		NR1D2		
		Low	High	*p* ^a^
**All patients**	**No**	115 (60.8)	74 (39.2)	0.031
	**Yes**	11 (39.3)	17 (60.7)	
**3 years after disease diagnosis**	**No**	117 (60.9)	75 (39.1)	0.015
	**Yes**	7 (33.3)	14 (66.7)	
**5 years after disease diagnosis**	**No**	107 (61.5)	67 (38.5)	0.042
	**Yes**	11 (40.7)	16 (59.3)	

^a^ χ^2^ or Fisher’s exact tests.

**Table 9:** Results of logistic regression for LR within 3 and 5 years after disease diagnosis.

3 Years after Disease Diagnosis			
Independent Variables		OR ^a^ [95% CI ^b^]	*p*
**Intercept**			<0.001
**Age**	(>71 vs ≤71)	1.48 [0.48, 4.59]	0.500
**Gender**	(man vs. woman)	1.64 [0.58, 4.60]	0.349
**T stage**	(3 + 4 vs. 1 + 2)	3.21 [0.38, 27.42]	0.286
**N stage**	(1 + 2 vs. 0)	1.29 [0.45, 3.71]	0.632
**Adjuvant Therapy**	(Yes vs. No)	3.59 [0.85, 15.01]	0.081
**NR1D2**	(High vs. Low)	3.04 [1.13, 8.15]	0.021
**5 years after disease diagnosis**			
**Independent variables**		**OR [95% CI]**	***p***
**Intercept**			<0.005
**Age**	(>71 vs ≤ 71)	1.44 [0.52, 3.98]	0.477
**Gender**	(man vs. woman)	1.12 [0.47, 2.69]	0.794
**T stage**	(3 + 4 vs. 1 + 2)	4.16 [0.50, 34.36]	0.186
**N stage**	(1 + 2 vs. 0)	1.01 [0.39, 2.64]	0.980
**Adjuvant Therapy**	(Yes vs. No)	2.48 [0.45, 8.27]	0.139
**NR1D2**	(High vs. Low)	2.45 [1.03, 5.85]	0.044

^a^ Odd Ratio; ^b^ Confidence Intervals.

**Table 10:** Multivariate Cox regression analysis of CRY1 and BMAL1 expression and clinicopathologic variables predicting survival in our cohort of CRC.

		OS		DFS	
Independent Variable		HR [95% CI]	*p* ^a^	HR [95% CI]	*p* ^a^
**Age**	(>71 vs ≤71)	1.023 [0.99, 1.06]	0.174	1.019 [0.99, 1,05]	0.248
**Gender**	(man vs. woman)	1.93 [1.02, 3.67]	0.044	1.793 [0.95, 3.93]	0.073
**TNM stage**	(III + IV vs. I + II)	2.35 [1.09, 5.07]	0.029	2.30 [1.07, 4.97]	0.033
**Adjuvant Therapy**	(Yes vs. No)	2.42 [0.94, 6.17]	0.066	2.56 [1.00, 6.58]	0.050
**CRY1**	(High vs. Low)	3.15 [1.15, 6.45]	0.023	2.90 [1.22, 6.85]	0.015
**BMAL1**	(High vs. Low)	0.52 [0.20, 0.97]	0.039	0.54 [0.26, 0.99]	0.048

^a^ Multivariate Cox regression analysis including age, gender, TNM stage, adjuvant therapy, CRY1 and BMAL1 proteins expression status.

## Supplementary Materials

https://www.mdpi.com/article/10.3390/biomedicines9080967/s1, Table S1: IHC staining conditions for each core circadian protein analysed, Table S2: Relationship between expression levels of circadian proteins and development of MM after disease diagnosis of individuals included in the study, Table S3: Relationship between expression levels of circadian proteins and development of LR after disease diagnosis of patients included in the study.
