# Supplementary material for: Core Circadian Clock Proteins as Biomarkers of Progression in Colorectal Cancer
Source: Biomedicines. 2021 Aug 6;9(8):967. doi: 10.3390/biomedicines9080967 (PMC8391187; doi:10.3390/biomedicines9080967)
Supplement: Supplementary file 1 [file biomedicines-09-00967-s001.zip › biomedicines-1299128-supplementary.pdf]

Supplementary Material

Table S1. IHC staining conditions for each core circadian protein analysed.

Protein	Conditions	
	**Antigen Retrieval**	**Primary Antibody**
CRY-1	CC1 [a] (pH 8.4, 24 min)	30 min, 37 °C
CRY-2	CC1 [a] (pH 8.4, 1 h)	32 min, 37 °C
PER-1	CC1 [a] (pH 8.4, 48 min)	1 h, 37 °C
PER-2	Ribo CC o CC2 [b] (pH 6, 24 min)	1 h, 37 °C
PER-3	Ribo CC o CC2 [b] (pH 6, 24 min)	1/50, 1 h, 37 °C
Bmal1	CC1 [a] (pH 8.4, 24 min), (1/50,)	30 min, 37 °C
NR1D2	CC1 [a] (pH 8.4, 24 min)	1 h, 37 °C

[a] CC1: ethylenediaminetetraacetic acid (EDTA)-based heat-induced antigen retrieval; [b] Ribo CC o CC2, citrate-based heat-induced enzyme retrieval.

Table S2. Relationship between expression levels of circadian proteins and development of MM after disease diagnosis of individuals included in the study.

		PER1			PER2			PER3			CRY2			BMAL1			NR1D2		
		Low	High	*p* [a]	Low	High	*p* [a]	Low	High	*p* [a]	Low	High	*p* [a]	Low	High	*p* [a]	Low	High	*p* [a]
**All patients**	**No**	59 (37.8)	97 (62.2)	ns	52 (33.3)	104 (66.7)	ns	142 (90.4)	15 (9.6)	ns	12 (7.6)	145 (92.4)	ns	21 (13.5)	134 (86.5)	ns	92 (59.0)	64 (41.0)	ns
	**Yes**	17 (43.6)	22 (56.4)		16 (40.0)	24 (60.0)		38 (90.5)	4 (9.5)		2 (4.8)	39 (95.2)		6 (15.0)	34 (85.0)		21 (52.5)	19 (47.5)	
**3 years after disease diagnosis**	**No**	60 (37.0)	102 (63.0)	ns	54 (33.1)	109 (66.9)	ns	147 (89.6)	17 (10.4)	ns	12 (7.3)	152 (92.7)	ns	20 (12.3)	142 (87.7)	ns	95 (58.3)	68 (41.7)	ns
	**Yes**	14 (48.3)	15 (51.7)		12 (41.4)	17 (58.6)		29 (93.5)	2 (6.5)		0 (0.0)	30 (100.0)		6 (20.7)	23 (79.3)		16 (55.2)	13 (44.8)	
**5 years after disease diagnosis**	**No**	56 (37.6)	93 (62.4)	ns	50 (33.6)	99 (66.4)	ns	133 (88.7)	17 (11.3)	ns	11 (7.3)	139 (92.7)	ns	19 (12.7)	130 (87.2)	ns	88 (59.1)	61 (40.9)	ns
	**Yes**	15 (42.9)	20 (57.1)		15 (41.7)	21 (58.3)		36 (94.7)	2 (5.3)		1 (2.7)	36 (97.3)		6 (16.7)	30 (83.3)		20 (55.6)	16 (44.4)	

[a] χ^2 or Fisher's exact tests.

Table S3. Relationship between expression levels of circadian proteins and development of LR after disease diagnosis of patients included in the study.

		PER1			PER2			PER3			CRY1			CRY2			BMAL1		
		Low	High	*p* [a]	Low	High	*p* [a]	Low	High	*p* [a]	Low	High	*p* [a]	Low	High	P[a]	Low	High	*p* [a]
**All patients**	**No**	73 (38.8)	115 (61.2)	ns	69 (36.5)	120 (63.5)	ns	171 (90.0)	19 (10.0)	ns	58 (30.7)	131 (69.3)	ns	14 (7.4)	176 (92.6)	ns	30 (16.0)	158 (84.0)	ns
	**Yes**	12 (42.9)	16 (57.1)		12 (42.9)	16 (57.1)		29 (100.0)	0 (0.0)		6 (20.7)	23 (79.3)		1 (3.4)	28 (96.6)		3 (10.7)	25 (89.3)	
**3 years after disease diagnosis**	**No**	75 (39.5)	115 (60.5)	ns	69 (35.9)	123 (64.1)	ns	173 (89.6)	20 (10.4)	ns	59 (30.7)	133 (69.3)	ns	15 (7.8)	178 (22.2)	ns	27 (14.1)	164 (85.9)	ns
	**Yes**	9 (40.9)	13 (59.1)		10 (47.6)	11 (52.4)		22 (100.0)	0 (0.0)		4 (18.2)	18 (81.8)		0 (0.0)	22 (100.0)		3 (14.3)	18 (85.7)	
**5 years after disease diagnosis**	**No**	69 (39.7)	105 (60.3)	ns	64 (36.3)	111 (63.7)	ns	155 (88.6)	20 (11.4)	ns	55 (31.4)	120 (68.6)	ns	14 (8.0)	162 (92.0)	ns	26 (14.9)	148 (85.1)	ns
	**Yes**	11 (40.7)	16 (59.3)		13 (48.1)	14 (51.9)		28 (100.0)	0 (0.0)		7 (25.0)	21 (75.0)		1 (3.6)	27 (96.4)		3 (11.1)	24 (88.9)	

[a] χ^2 or Fisher's exact tests.
